# The Implications of Socioeconomic Status by ZIP Code on Maternal-Fetal Morbidity and Mortality in San Antonio, Texas

**DOI:** 10.7759/cureus.54636

**Published:** 2024-02-21

**Authors:** Vaishnavi J Patel, Victoria Delano, Aishwarya Juttu, Huraiya Adhora, Aroob Zaheer, Leticia Vargas, Blaine Jacobs

**Affiliations:** 1 Office of Research and Innovation, University of the Incarnate Word School of Osteopathic Medicine, San Antonio, USA; 2 Department of Obstetrics and Gynecology, Metropolitan Methodist Hospital, San Antonio, USA; 3 Department of Obstetrics and Gynecology, University of the Incarnate Word School of Osteopathic Medicine, San Antonio, USA; 4 Department of Pharmacology, University of the Incarnate Word School of Osteopathic Medicine, San Antonio, USA

**Keywords:** san antonio, healthcare resources, healthcare quality, racial disparities, risk factors, healthcare access, zip code disparities, socioeconomic status, fetal mortality, maternal mortality

## Abstract

Introduction

Over the past 20 years, the number of pregnancy-related fatalities in the United States has been on the rise. Increases in maternal and fetal mortality have been attributed to low socioeconomic status (SES). This raises the question of whether all geographical locations are proportionally affected by this upward trend in pregnancy-related fatalities. San Antonio is one of the largest cities in the United States and is known for its economic segregation. This study aims to compare the maternal and fetal health outcomes of mothers from diverse socioeconomic backgrounds in San Antonio, Texas.

Methods

To analyze the relationship between pregnancy-related mortality rates and SES in San Antonio, Texas, the International Classification of Diseases (ICD)-10 codes for maternal and fetal demise and their associated risk factors were identified. The ICD-10 codes were used to compare the health outcomes of pregnant women from the highest SES ZIP Code (78255, median income $124,397) to women from the lowest SES ZIP Code (78207, median income $25,415) using the Texas Inpatient Public Use Data File for 2016, which contains information on 93-97% of all hospital discharges in San Antonio, Texas.

Results

Notably, pregnant women from the high SES ZIP Code were admitted to the hospital from clinics or a physician’s office (68.8%), while pregnant women from the low SES ZIP Code were admitted to the hospital from non-healthcare facilities like home or workplace (62.5%). In addition, a greater percentage of patients from the low SES ZIP Code were Black (4.3% vs 1.3%) or Hispanic (88.5% vs 35.1%). Compared to women from the high SES ZIP Code, women from the low SES ZIP Code experienced more fetal deaths and a higher prevalence of maternal and fetal risk factors such as obesity (47.6% vs 32.5%), asthma (1.7% vs 1.3%), hypertension (0.8% vs 0%), substance abuse (0.5% vs 0%), diabetes mellitus (9.8% vs 7.8%), preeclampsia (7.7% vs 2.6%), and multiple C-sections (35.5% vs 28.6%). Finally, fetal mortality rates were higher in the low SES ZIP Code (1.1% vs 0%). Although there were no statistically significant maternal or fetal mortality differences between the ZIP Codes, the trend suggests that women’s health outcomes in San Antonio are not equitable.

Discussion

Analysis reveals disproportionate health outcomes for women in south San Antonio. Further investigation is warranted to better understand the role social and medical factors play in these results. Investigating the relationship between SES and pregnancy-related mortality can help to better inform healthcare providers and identify ways to improve women’s health outcomes in San Antonio, Texas.

## Introduction

Advances in medicine and living standards have significantly lowered the rate of maternal mortality. However, maternal and fetal mortality remains a concern in the United States [1]. Over the past 20 years, the number of pregnancy-related fatalities in the United States has been on the rise [1]. This raises the question of whether all social classes are proportionally affected by this upward trend and whether geographical location is a variable contributing to increased mortality. Texas is one of the largest states in the United States with San Antonio being its second largest city with a population of 1.53 million. Investigating the relationship between socioeconomic status (SES) and pregnancy-related mortality in San Antonio, where maternal mortality rates vary depending on the geographic locations within the city, can be instrumental in comprehending the rising trend.

Importance of discussing SES

SES can be defined as a measure of an individual’s economic assets in combination with their social status. It is a commonly accepted notion that increases in SES mirror increases in overall health outcomes, while a lower SES is a risk factor for major causes of premature mortality-including maternal mortality. SES is commonly measured through education level, yearly income, and occupational status [2]. Educational level indicates the level of formal education an individual has attained, serving as a foundation for skills, knowledge, and career opportunities [2]. Yearly income reflects the financial resources available to a person or family, influencing their access to healthcare, housing, and other essential needs [2]. Occupational status highlights the type of work an individual engages in and their corresponding health issues and social standing [2]. These three components are interrelated and often used collectively to create a more comprehensive picture of an individual's or a family's SES. People with higher levels of education, higher incomes, and prestigious occupations can be generally considered to have a higher SES. These three measures work in combination to create a gradient between an individual’s SES and the quality of healthcare they receive. Previous research has shown a direct relationship between SES and pregnancy-associated complications such as preeclampsia, eclampsia, and maternal death [3-4]. These complications are related to poor nutrient intake, inadequate prenatal care, and increased barriers to obtaining maternal health services.

Importance of maternal mortality

The World Health Organization (WHO) defines maternal mortality as “the death of a woman while pregnant or within 42 days of termination of pregnancy, irrespective of the cause of death (obstetric and non-obstetric)” which includes unintentional/accidental and incidental causes [5]. Among developed nations, the United States has one of the highest rates of maternal mortality with about 18.0 deaths per 100,0000 live births [5]. In 2015, the maternal mortality rate in Texas was 19.7 per 1,000 delivered. In the United States, 861 women died from causes of maternal mortality by 2020, a value that increased from 754 in 2019 and 658 in 2018, indicating a rise in maternal mortality [5-6]. Texas accounts for nearly 10% of all births in the United States yearly and, as a result, the increased rates of maternal mortality in Texas have national implications [7]. It is important to look at San Antonio specifically because maternal mortality is likely a worse problem than we realize in South Texas. Compared to national mortality rates, the effect in South Texas is likely underestimated as San Antonio has greater rates of diabetes and cardiovascular disease, which are factors contributing to increased maternal mortality. Moreover, the population may require novel or specific interventions unique to San Antonio.

Importance of fetal mortality

The CDC defines fetal mortality as the “spontaneous intrauterine death of a fetus at any time during pregnancy” while stillbirth is referred to as “fetal death at 20 weeks of gestation or more” [8]. In the United States, about one million fetal deaths and stillbirths are reported annually [9]. Embryonic and fetal mortality is caused by a variety of factors throughout the course of gestation which are related to maternal health (diabetes, syphilis, sickle cell disease, HIV, viral hepatitis, etc.), labor/delivery management (intrapartum hemorrhage, cord complications, home deliveries, etc.), and socioeconomic factors [10]. Previous studies have explored the inverse association between infant mortality and SES in several countries including the United States to emphasize the importance of adequate access to healthcare during pregnancy for fetal development and monitoring. This project will continue to extend that research in San Antonio specifically to provide more accurate and population-tailored estimates of health disparities.

San Antonio

The three pillars of SES include wealth, education, and occupation. Nationally, it has been shown that maternal mortality correlates with SES. We used variables that represent the SES pillars such as median household income and area-based ZIP (Zone Improvement Plan) Codes in San Antonio due to their measurable characteristics to determine SES’s correlation with maternal mortality outcomes. In 2020, the total population for San Antonio, Texas, was 1.53 million making the city one of the largest in the country [9]. As a result, maternal outcomes in San Antonio will have a greater national impact. Research has shown that between 2011 and 2016 the overall rates of maternal morbidity have remained between 17.0 per 1,000 delivered to 19.9 per 1,000 deliveries [7]. In San Antonio, the ZIP Codes 78112, 78201, 78207, 78210, 78211, 78214, 78221, 78225, 78228, 78237, 78242, and 78252 have had maternal morbidity rates at or above the 75th percentile across three years. While the ZIP Code 78015 has had a maternal morbidity rate at or below the 25th percentile across three years [7]. These statistics show that overall rates of maternal mortality vary greatly between geographic locations in San Antonio. Bexar County residents south of Hildebrand Avenue on a City Council district map have a higher poverty rate, lower educational attainment, and 15-20% lower life expectancy than those who live north of that line [9].

Use of ZIP Codes for measuring SES

Prior research indicates that area-based indicators such as median household income and ZIP Code are the recommended measures to track health outcomes based on SES due to ease of use, consistency, and completeness [8]. According to the 2020 United States Census, the median household income in San Antonio is $53,420 (2016-2020). This value, however, differs greatly between San Antonio’s neighborhoods. The median income in the west, inner east, and southside neighborhoods with the ZIP Codes 78219, 78220, 78207, 78225, and 78204 is $24,744-$35,000. Among this population, 25-39% of families are living below the poverty level [11]. Inversely, the median income of neighborhoods along far west, northcentral with the ZIP Codes 78253, 78256, 78257, 78258, 78260, 78255, 78259, 78261, and 78266 is $93,000-$122,706, which is double the average income of the city. Additionally, when compared to the ZIP Codes with the lowest income, only five or fewer families in this area are living below poverty [11]. In pregnant women, low SES can increase the risk of adverse pregnancy outcomes [4,12]. Research has established relationships between low SES and maternal health and birth outcomes across the world as well as across states within the United States [4,13]. It was further found that environments characterized by higher SES, where women thrive socially, financially, and politically, tend to be associated with more favorable health conditions for women along with lower levels of stress, which contribute to more favorable birth outcomes, lower pregnancy-related complications such as abortion, preterm delivery, preeclampsia, eclampsia, and gestational diabetes, and lower rates of infant mortality compared with environments characterized by lower SES [4,12].

Several factors such as marital or partner status, level of education, adequate prenatal care, and social support, play a major role in pregnancy outcomes and can be a buffer for the adverse associations of lower SES [12].

Study purpose

The purpose of this study is to determine whether there is a disparity between maternal and fetal mortality based on SES using ZIP Codes in San Antonio, Texas. We hypothesize that there is an inverse relationship between SES based on ZIP Codes and maternal-fetal mortality rate in San Antonio. To determine if there is a relationship between ZIP Code in San Antonio and maternal-fetal mortality rate we determined the median household incomes for ZIP Codes in San Antonio and compared their maternal and fetal mortality rates and risk factors. The findings of this project can be used to understand what factors are responsible for the increase in maternal-fetal mortality trend on a national level and to improve health outcomes for mothers in San Antonio.

## Materials and methods

In this retrospective cohort study, we used the Texas Inpatient Public Use Data File (PUDF) to compare mortality in pregnant women and their fetuses across ZIP Codes within San Antonio, Texas, for all four quarters of the year 2016. International Classification of Disease (ICD) codes were used to identify a maternal or fetal demise. In addition, the maternal and fetal outcomes were compared between the highest and lowest socioeconomic ZIP Codes within San Antonio. The study was approved by the University of the Incarnate Word Institutional Review Board (approval number: 2023-1300-EXP-v1).

ICD-10 codes

The ICD-10 codes for maternal and fetal demise as well as maternal and fetal risk factors were searched on the National Center for Health Statistics - ICD⁠-⁠10⁠-⁠CM search engine on the Centers for Disease Control and Prevention (CDC) portal for fiscal year 2023 (October 1, 2022, to September 30, 2023) [14]. Keywords used for the search for maternal demise included “pregnancy”, “death”, “maternal”, or “maternal death”. An inclusion criteria was set based on the maternal death defined by the World Health Organization (WHO). The search findings were then reviewed by two authors to filter for the inclusion criteria which yielded codes P01.6 and R99. A list of the most common risk factors for maternal demise was obtained from the National Institutes of Health (NIH) which reported obesity, asthma, compromised immune system, systemic diseases, coronary vascular disease (CVD), old age, tobacco use, twins/triplets, diabetes mellitus, preeclampsia, and previous history of cesarean section [15]. Similarly, these phrases were included in the search and filtered for codes that pertained to pregnancy. The list included codes O99.21, O99.5, O99.1, O16, O99.4, O09.52, O09.51, O99.33, O30.9, O31.2, O24.419, O24.91, O14.9, and O34.219.

Keywords used for the search for fetal demise included “pregnancy”, “death”, “fetal”, or “fetal death”. An inclusion criteria was set based on the fetal death defined by WHO. The search findings were then reviewed by two authors to filter for the inclusion criteria which yielded codes O02.1, O03, O03.9, O03.89, O02.1, O04, O00, O05, P95, O31.2, and O36.4. A list of the most common risk factors for fetal demise was obtained by NIH to include small fetal size, maternal medical conditions of obesity, diabetes, and hypertension, as well as a maternal history of multiple gestation, tobacco use, substance use, and older age [16]. These phrases were included in the search and filtered for codes that pertained to pregnancy. The list included codes O99.21, O16, O24.419, O24.91, O30.9, O31.2, O99.33, O99.32, O99.31, P05.9, O36.59, O09.52, and O09.51 (Table 1).

**Table 1:** ICD-10 Codes for Risk Factors Related to Maternal or Fetal Demise That Were Utilized in the Study

Demise	ICD-10 Code	Risk Factor
Maternal	O99.21	Obesity complicating pregnancy, childbirth, and the puerperium
	O99.5	Diseases of the respiratory system complicating pregnancy, childbirth, and the puerperium
	O99.1	Other diseases of the blood and blood-forming organs and certain disorders involving the immune mechanism complicating pregnancy, childbirth, and the puerperium
	O16	Hypertension complicating pregnancy
	O99.4	Diseases of the circulatory system complicating pregnancy, childbirth, and the puerperium
	O09.52	Pregnancy supervision of elderly mother multigravida
	O09.51	Pregnancy supervision of elderly mother primigravida
	O99.33	Tobacco complicating pregnancy
	O30.9	Multiple gestation
	O31.2	Pregnancy complicated by multiple gestation
	O24.419	Gestational Diabetes
	O24.91	Pregnancy complicated by diabetes
	O14.9	Pregnancy complicated by preeclampsia
	O34.219	Vaginal delivery following previous cesarean delivery
Fetal	O99.21	Obesity complicating pregnancy, childbirth, and the puerperium
	O16	Hypertension complicating pregnancy
	O24.419	Gestational diabetes
	O24.91	Pregnancy complicated by diabetes
	O30.9	Multiple gestations
	O31.2	Pregnancy complicated by multiple gestation
	O99.33	Tobacco complicating pregnancy
	O99.32	Pregnancy complicated by drug use
	O99.31	Pregnancy complicated by alcohol use
	P05.9	Newborn affected by fetal growth retardation
	O36.59	Pregnancy complicated by fetal growth retardation

ZIP Code methods 

San Antonio remains one of the most economically segregated cities in the country, with those living north and northwest of downtown having more economic prosperity, higher education beyond a high school degree, and better health outcomes such as less prevalence of diabetes, hypertension, and heart disease. Those who live in central San Antonio and areas immediately east, west, and south of the city center experience higher rates of poverty, disease, and lower educational attainment [11,12]. Pregnant women are among the vulnerable populations that are often affected by the gap in healthcare seen between SES. Prior studies have shown that low SES is associated with increased pregnancy complications such as abortion, preterm delivery, preeclampsia, eclampsia, and gestational diabetes [4]. Additionally, women with lower SES are less likely to receive prenatal care; inadequate prenatal care is associated with poor obstetric outcomes such as preterm delivery, preeclampsia, and stillbirths [4,11,12].

According to the Distressed Communities Index (DCI), 16 San Antonio ZIP Codes rank in the 80th percentile for the nation’s most distressed communities. ZIP Codes 78207, 78208, 78204, and 78207, capturing much of the urban Westside, scored in the 98th percentile of all ZIP Codes analyzed in the study. These findings highlight the extent of San Antonio’s economic segregation and mirror those of median household income.

This study utilized median household income to measure SES due to its consistency and completeness compared to other factors. The ZIP Codes determined to have the lowest household incomes are 78219, 78220, 78207, 78225, 78204, and 78208. These ZIP Codes have an average household income of $24,744-$35,000. On the other hand, the ZIP Codes with the highest household incomes are 78253, 78256, 78257, 78258, 78260, 78255, 78259, 78261, and 78266. These ZIP Codes have an average household income of $93,000-$122,706. Of the two groups, the ZIP Code with the lowest average household income is 78207, with a median income of $25,415, and the ZIP Code with the highest average household income is 78255, with a median income of $124,397. This data is supported by the Individual Income Tax ZIP Code Data provided by the Internal Revenue Service (IRS). For the year 2020, the ZIP Code 78207 had a majority of its population file tax returns that fell into the adjusted gross income range of $1-$25,000. The ZIP Code 78255 had a majority of its population file tax returns that fell into the adjusted gross income range of $100,000-200,000. These two ZIP Codes were utilized to highlight the gap in healthcare seen among different SESs [17].

Data source

The Texas Inpatient Public Use Data File (PUDF) for all quarters of the year 2016 was used to perform a retrospective, population-based cohort study. This database consists of discharge data on all patients admitted to licensed hospitals in Texas with some exclusions. Some hospitals are statutorily exempt from the reporting requirements, such as (a) those located in a county with a population of less than 35,000, (b) those with fewer than 100 licensed hospital beds and located in a rural area, (c) those that do not seek government reimbursement, and (d) federal hospitals. However, despite these exclusions, the database still accounts for 93-97% of all hospital discharges in Texas [13].

Patient population

The following ICD-10 codes were utilized as the inclusion criteria for these findings. Maternal demise utilized ICD-10 codes: P01.6 and R99. Fetal demise utilized ICD 10: O02.1, O03, O03.9, O03.89, O02.1, O04, O00, O05, P95, O31.2, and O36.4. This inclusion criteria helped us identify pregnant women who experienced demise during pregnancy or fetal demise that occurred in an inpatient setting. We also examined potential risk factors for maternal or fetal demise using their relevant ICD-10 codes. Until the transition to ICD-10 codes in the third quarter of 2015, ICD-9 codes were used to identify maternal and fetal demise. Due to this, only the year 2016 was utilized in the analysis due to its utilization of the more updated ICD-10 codes.

PUDF reports discharge information rather than patient information, meaning that readmissions were not accounted for within the data. Prior research has shown that advanced maternal age and teen pregnancies are associated with increased maternal mortality [13,18]. As a result, our study focused on women in the child-bearing age (20-34 years). ZIP Codes were used to classify patients as being from San Antonio and further work was done to determine the relative SES of certain ZIP Codes. The highest and lowest SES were the focus of our analysis.

Data analysis

The difference between maternal mortality and fetal mortality in the highest socioeconomic ZIP Code and the lowest socioeconomic ZIP Code was measured using a two-tailed t-test where a probability value of less than 0.05 was considered statistically significant for quantitative variables. Quantitative variables were summarized using mean and standard deviation (SD). Categorical variables were described using frequencies and percentages. Significance for qualitative variables was calculated using a chi-squared test where a probability value of less than 0.05 was considered statistically significant. All analysis was performed using Microsoft Excel (Microsoft Corporation, Redmond, Washington, United States).

Unfortunately, a limitation of data interpretation was that deaths related to drug or alcohol abuse and/or chronic comorbid conditions could not be excluded due to their relationship with SES. For situations where an ICD code was vague and could not be classified in one category, these hospitalizations were excluded from the analysis. These results were not utilized due to the smaller sample size inhibiting the ability to calculate an accurate maternal or fetal mortality rate.

## Results

Prior analysis showed that the ZIP Code with the lowest SES in San Antonio was 78207 and the ZIP Code with the highest SES was 78255. The summary data for women living in these ZIP Codes who were in the age group of 20-34 years and admitted to the Obstetrical Unit are given in Table 2.

**Table 2:** Summary Data for Hospitalized Pregnant Women in San Antonio by ZIP Code in 2016 78207 indicates the ZIP Code with the lowest SES in San Antonio, Texas (median income $25,415) and 78255 indicates the ZIP Code with the highest SES in San Antonio, Texas (median income $124,397). *For the Type of Admission, Urgent implies a need for timely attention, but the situation is not immediately life-threatening, while Emergency indicates a critical and potentially life-threatening situation that demands immediate action. **For Source of Admission, a non-healthcare facility is defined as any location or institution that does not offer healthcare services such as a home, shelter, social service agencies, correctional facilities, schools/universities, workplaces, recreational facilities, religious institutions, government offices, or crisis intervention centers. Transfer indicates patients being moved from one healthcare-providing facility such as a hospital to another. This allows continuity of care based on facility resources, bed space, and other factors. SES: socioeconomic status

		78207 (low SES) (N=651, 89.4%), n (%)	78255 (high SES) (N=77, 10.6%), n (%)	Total (N = 728), n
Type of Admission*	Emergency	241 (37.0%)	20 (26.0%)	261
	Urgent	22 (3.4%)	7 (9.1%)	29
	Elective	363 (55.8%)	50 (64.9%)	413
	Trauma Center	1 (0.2%)	0 (0.0%)	1
Source of Admission**	Non-Healthcare Facility	407 (62.5%)	24 (31.2%)	431
	Clinic or Physician's Office	241 (37.0%)	53 (68.8%)	294
	Transfer	2 (0.3%)	0 (0.0%)	2
	Court/Law Enforcement	1 (0.2%)	0 (0.0%)	1
Race	American Indian /Eskimo/Aleut	0 (0.0%)	0 (0.0%)	0
	Asian or Pacific Islander	0 (0.0%)	5 (6.5%)	5
	Black	28 (4.3%)	1 (1.3%)	29
	White	435 (66.8%)	53 (68.8%)	488
	Other	188 (28.9%)	18 (23.4%)	206
Ethnicity	Hispanic Origin	576 (88.5%)	27 (35.1%)	603
	Not of Hispanic Origin	75 (11.5%)	50 (64.9%)	125
Age	20-24 years	317 (48.7%)	3 (3.9%)	320
	25-29 years	219 (33.6%)	25 (32.5%)	244
	30-34 years	115 (17.7%)	49 (63.6%)	164

Notably, as seen in Table 2, in the low SES ZIP Code group, many of the admissions (62.5%) came from a non-healthcare facility such as the patient’s home, while the high SES ZIP Code saw more admissions (68.8%) from a clinic or physician’s office. In the low SES ZIP Code group, there was a slightly greater percentage of Black (4.3%) and Other races (28.9%). In addition, the low SES ZIP Code group had a much higher percentage of women of Hispanic origin (88.5%). When considering age, there was a greater percentage (48.7%) of younger mothers aged 20-24 years in the low SES ZIP Code group and a greater percentage (63.6%) of older mothers aged 30-34 years in the high SES ZIP Code group.

For the two ZIP Codes, there were two methods used to determine the number of maternal mortalities. Firstly, the Texas Inpatient PUDF lists the status of the patient as either Routine Discharge, Non-Routine Discharge, Left Against Medical Advice, Expired, or Still Patient (Table 3). Since the dataset we used consisted of only young women admitted to the Labor & Delivery units, it is reasonable to assume that a status of “Expired” indicated patients who passed away during their time in that unit due to the other deadly diagnostic ICD-10 codes these women had. This method came with no women having passed away in either the low SES or the high SES ZIP Codes. The other method used to determine if there were maternal mortalities was using the ICD-10 codes for Newborns affected by maternal death (P01.1) and Death of unknown cause (R99), which also showed no maternal mortalities between the two ZIP Codes. Notably, there were more non-routine discharges and patients who left against medical advice in the low SES ZIP Code than in the high SES ZIP Code. Though no maternal deaths were seen when comparing these ZIP Codes, maternal deaths were detected in other ZIP Codes in Texas.

**Table 3:** Maternal Mortality for Hospitalized Pregnant Women in San Antonio by ZIP Code in 2016 78207 indicates the lowest SES ZIP Code in San Antonio, Texas (median income $25,415) and 78255 indicates the highest SES ZIP Code in San Antonio, Texas (median income $124,397). SES: socioeconomic status

		78207 (low SES) (N = 651, 89.4%), n (%)	78255 (high SES) (N = 77, 10.6%), n (%)	Total (N = 728), n
Patient Status	Routine Discharge	643 (98.8%)	77 (100.0%)	720
	Non-Routine Discharge	5 (0.8%)	0	5
	Left Against Medical Advice	2 (0.3%)	0	2
	Admitted	1 (0.2%)	0	1
	Expired	0	0	0
	Still Patient	0	0	0
Newborns affected by maternal death		0	0	0
Death by unknown cause		0	0	0
	Total Maternal Deaths	0	0	0

In the low SES ZIP Code group, there were a total of seven fetal deaths (Table 4). Five of them were from spontaneous abortion while two were from ectopic pregnancy. In the high SES ZIP Code, there were no fetal deaths. The p-value for fetal deaths was p = 0.3605, which means there was no association between ZIP Code and fetal mortality.

**Table 4:** Fetal Mortality for Hospitalized Pregnant Women in San Antonio by ZIP Code in 2016 The association between the low and high SES ZIP Codes was examined using p-values calculated through the chi-square test. 78207 indicates the lowest SES ZIP Code in San Antonio, Texas (median income $25,415) and 78255 indicates the highest SES ZIP Code in San Antonio, Texas (median income $124,397). SES: socioeconomic status

Fetal Mortality ICD-10 Descriptions	78207 (low SES) (N=651, 89.4%), n (%)	78255 (high SES) (N=77, 10.6%), n (%)	Total (N=728), n (%)	p-values
Fetal death from unspecified cause	0	0	0	
Missed abortion	0	0	0	
Spontaneous abortion	5 (0.8%)	0	5 (0.7%)	0.44
Complete or unspecified spontaneous abortion without complication	0	0	0	
Complete or unspecified spontaneous abortion with other complications	0	0	0	
Early fetal death	0	0	0	
Medical abortion	0	0	0	
Ectopic pregnancy	2 (0.3%)	0	2 (0.3%)	0.626
Other abortion	0	0	0	
Continuing pregnancy after intrauterine death of one fetus or more	0	0	0	
Maternal care for intrauterine death	0	0	0	
Total Fetal Deaths	7 (1.1%)	0	7 (1.0%)	0.361

As seen in Table 5, there were many more risk factors that affected the women in the low SES ZIP Code and not as much in the high SES ZIP Code. Of the women in the low SES ZIP Code, 47.6% experienced obesity compared to 32.5% who experienced it in the high SES ZIP Code. Similarly, the percentages for asthma, hypertension, smoking, diabetes mellitus, preeclampsia, and previous cesarian section history were slightly higher in the low SES ZIP Code than in the high SES one.

**Table 5:** Maternal Risk Factors for Hospitalized Pregnant Women in San Antonio by ZIP Code in 2016 The association between the low and high SES ZIP Codes was examined using p-values calculated through the chi-square test. 78207 indicates the lowest SES ZIP Code in San Antonio, Texas (median income $25,415) and 78255 indicates the highest SES ZIP Code in San Antonio, Texas (median income $124,397). SES: socioeconomic status; CVD: cardiovascular disease

Maternal Risk Factors	78207 (low SES) (N=651, 89.4%), n (%)	78255 (high SES) (N=77, 10.6%), n (%)	Total (N=728), n (%)	p-values
Obesity	310 (47.6%)	25 (32.5%)	335 (46.0%)	0.012
Asthma	11 (1.7%)	1 (1.3%)	12 (1.7%)	0.799
Immunocompromise	0	0	0	
Hypertension	4 (0.6%)	0	4 (0.6%)	0.49
	1 (0.2%)	0	1 (0.1%)	0.731
CVD	0	0	0	
Smoking	3 (0.5%)	0	3 (0.4%)	0.551
Multigravida	5 (0.8%)	2 (2.6%)	7 (1.0%)	0.12
	0	0	0	
Diabetes Mellitus	59 (9.1%)	6 (7.8%)	65 (8.9%)	0.712
	0	0	0	
	1 (0.2%)	0	1 (0.1%)	0.731
	3 (0.5%)	0	3 (0.4%)	0.551
Pre-Eclampsia	50 (7.7%)	2 (2.6%)	52 (7.1%)	0.102
Previous Cesarian Section	231 (35.5%)	22 (28.6%)	253 (34.8%)	0.228
Total Maternal Risk Factors, n	678	58	736	

The trends seen in the maternal risk factors are also the same for the fetal risk factors (Table 6). The exception was that 15.6% of the mothers in the high SES ZIP Code group experienced some form of fetal growth retardation, while the mothers in the low SES ZIP Code group only experienced fetal growth retardation at a percentage of 8.4% (p-value = 0.0527).

**Table 6:** Fetal Risk Factors for Hospitalized Pregnant Women in San Antonio by ZIP Code in 2016 The association between the low and high SES ZIP Codes was examined using p-values calculated through the chi-square test. 78207 indicates the lowest SES ZIP Code in San Antonio, Texas (median income $25,415) and 78255 indicates the highest SES ZIP Code in San Antonio, Texas (median income $124,397). SES: socioeconomic status; CVD: cardiovascular disease

Fetal Risk Factors	78207 (low SES) (N=651, 89.4%), n (%)	78255 (high SES) (N=77, 10.6%), n (%)	Total (N=728), n (%)	p-values
Obesity	310 (47.6%)	25 (32.5%)	335 (46.0%)	0.012
Asthma	11 (1.7%)	1 (1.3%)	12 (1.7%)	0.799
Immunocompromise	0	0	0	-
Hypertension	5 (0.8%)	0	5 (0.7%)	0.49
CVD	0	0	0	-
Substance Abuse	3 (0.5%)	0	3 (0.4%)	0.551
Multigravida	5 (0.8%)	2 (2.6%)	7 (1.0%)	0.12
Diabetes Mellitus	53 (9.8%)	6 (7.8%)	69 (9.4%)	0.712
Small Fetal Size	55 (8.4%)	12 (15.6%)	67 (9.2%)	0.018
Total Fetal Risk Factors, n	455	46	501	

## Discussion

Our analysis found no maternal mortalities in either low or high SES ZIP Codes. However, disparities emerged in maternal-fetal risk factors and fetal mortality rates. The low SES ZIP Code exhibited more non-routine discharges, instances of patients leaving against medical advice, younger patient ages, and increased environmental and maternal health risk factors such as obesity, hypertension, diabetes, minority status, smoking history, and previous cesarian sections. These factors collectively contributed to a higher fetal mortality rate in the low SES ZIP Code group compared to women residing in the high SES ZIP Code. Notably, the majority of pregnant women from the high SES ZIP Code were admitted to the hospital from a clinic or physician's office, while the majority of lower SES pregnant women were admitted from a non-healthcare facility such as home, workplace, shelter, school, or other similar locations. This discrepancy may reflect differences in access to prenatal care and healthcare resources, contributing to distinct admission patterns based on SES. Furthermore, the results indicate that in San Antonio, women residing in low socioeconomic areas face a higher prevalence of maternal medical risk factors including obesity, asthma, hypertension, smoking, diabetes mellitus, preeclampsia, and previous cesarian section history. Moreover, the odds of having three or more risk factors were 1.5 times higher for women from the low SES ZIP Code. This increased risk suggests that factors like epigenetics, the environment, local healthcare, and access to adequate support may contribute to increased pregnancy-related complications and fetal mortality.

Table 6 highlights a correlation between SES and fetal growth retardation, with an increased likelihood in mothers from the high SES ZIP Code compared to mothers from the low SES ZIP Code. The causes for the fetal growth retardation seen in the high SES population are nearly double those within the low SES population. This association can be attributed to the higher probability of older mothers residing in high SES areas, and requires further study. Advanced maternal age has been linked to an increased risk of medical conditions such as diabetes, high blood pressure, and the risk of fetal growth retardation. A study conducted by Oud et al. highlights a notable shift in the obstetric population, reflecting an increasing presence of older women with chronic comorbidities. This trend coupled with the growing prevalence of cesarean sections is believed to be significant contributors to the observed upward trend in fetal growth retardation risk [18].

Previous literature aligns with our findings, indicating a consistent trend where low SES populations experience higher maternal-fetal mortality rates and pregnancy-related complications. A 2018 study conducted in Korea found that pregnant women in the low SES group received inadequate prenatal care and were more likely to have pregnancy-related complications such as abortion (30.1%), undergo cesarean delivery (45.8%), and have preeclampsia (1.5%) compared to the middle/high SES group [19]. This trend supports our findings that the low SES ZIP Code in San Antonio experienced higher rates of cesarean delivery, preeclampsia, and spontaneous abortion. A study conducted by Peacock et al. looked at the effects of socioeconomic factors on preterm delivery (less than 37 weeks) which revealed that adverse social circumstances such as low social class, low education, marital status, poor housing conditions, and low income are significantly associated with preterm delivery [20]. This finding supports our hypothesis that there exists an inverse relationship between social factors specific to low SES and risk factors for maternal-fetal mortality. Furthermore, a study by Silva et al. found that low educational level is associated with the development of preeclampsia, substance abuse issues, financial issues, poor body mass index, poor blood pressure control, and unsatisfactory working conditions in pregnant women [21]. This association supports our finding that the low SES ZIP Code in San Antonio, 78207, experienced more maternal and fetal risk factors such as obesity, asthma, hypertension, substance abuse, diabetes, fetal macrosomia, and preeclampsia which could be correlated with higher fetal mortality in that ZIP Code.

Analysis of 2016 data collected in San Antonio, Texas, showed a statistically insignificant difference between maternal mortality and ZIP Code. This is an area of research that should be replicated and expanded to include more ZIP Codes and multiple years of patient data. When the inclusion criteria were expanded to the entirety of Texas in 2016, 15 maternal mortalities were seen in the population of 245,364 deliveries (see Appendix A). This is a state maternal mortality rate of one death per 4,230 live births, higher than the national average of one death per 5,555 live births [22]. This rate is consistent with other literature about the Texas maternal mortality rates. While our study showed no maternal mortality between the two ZIP Codes studied in 2016, the low SES ZIP Code group had a greater number of fetal deaths, possibly attributed to the prevalent social factors specific to low SES. A rise in maternal mortality, like that seen in Texas, can be associated with a higher prevalence of absence of high-school completion, attending less than 10 prenatal visits, and being of African American race [23]. Maternal and fetal health disparities are signs of deeper underlying social and economic injustices that need to be further investigated to provide adequate care to vulnerable populations.

Our study found no maternal mortalities in San Antonio in 2016 (see Appendix B), which is consistent with data from the CDC stating only six deaths per 1,000 births in 2016 [22]. The sample pool in 2016 had fewer births (n=671) with seven fetal deaths which indicates a tendency towards a higher rate of deaths per 1,000 births. By understanding the interplay of social and medical variables, healthcare leaders can be better informed and make conscious decisions that improve maternal and fetal outcomes proportionally across all social classes.

Benefits of using ICD codes

The ICD serves as the systematic coding of all diagnoses, symptoms, and procedures used by healthcare providers. ICD codes provide data on mortality and morbidity by tracking information on health maintenance and preventative care which can also allow for reimbursements from healthcare insurers through billing [24]. This can be helpful in addressing medical and epidemiologic concerns. However, this classification system can be difficult to use in situations where patient information is limited or unavailable as symptoms coded can have a variety of etiologies [25,26]. Overall, assuming ICD-10 codes were reported correctly, they offer a reliable diagnostic system for medical professionals. On the contrary, if there were incorrect diagnostic ICD-10 codes entered by healthcare providers, it would be difficult to identify the misdiagnosis with the dataset used since they were de-identified.

Limitations

ICD-10 codes have specific descriptions of diagnoses that are listed on the National Center for Health Statistics-ICD-10 search engine on the CDC portal [14]. The keywords used to search and isolate ICD-10 codes for maternal and fetal demise were set based on the definition established by WHO. This search strategy resulted in ICD-10 codes with diagnoses that were non-specific to the various etiologies of maternal and fetal demise. As a result, it yielded fewer than expected ICD-10 codes for maternal demise, which could have limited the results.

The use of the Texas Inpatient PUDF for 2016 is representative of 93-97% of all hospital discharges in Texas, which meant the results from the data analysis captured the majority of the San Antonio population who went to licensed hospitals. However, the sample size was limited due to the usage of data from only 2016 which posed a difficulty in yielding significant results. Since 2016 was the first year to transition from ICD-9 codes to ICD-10 codes, not all ICD-10 codes for pertinent risk factors such as hypertension were updated to the latest ICD-10 codes. This delay in the update posed a risk of not yielding accurate ICD-10 codes with their corresponding diagnoses and risk factors. Furthermore, this dataset excluded reports from counties with a population of less than 35,000, those with fewer than 100 licensed hospital beds, and those located in a rural area. In addition to these exclusions, the data used was also limited to in-patients in hospital settings, specifically those who were transferred to the obstetrics and gynecology unit, and does not account for alternative locations such as inpatients who were not transferred to obstetrics and gynecology, out-patient facilities, birthing centers, and home deliveries with midwives. Finally, given the focus of this analysis on San Antonio, Texas, the findings may not be generalizable to other regions or urban settings.

Recommendations

The future directions of this project can be based on the expansion of the limitations in this study. Using datasets from 2016-2023 will yield results that represent the latest ICD-10 codes and will increase the sample size which may provide stronger evidence for a significant association between SES and pregnancy-related mortality. Since the PUDF had exclusions mentioned previously, the usage of alternative data files that cover the exclusions would ensure that all counties within the low and high SES ZIP Codes in San Antonio are accounted for. The keywords used to search for ICD-10 codes can also be modified to better identify ICD-10 codes that are more specific for the various etiologies of maternal and fetal demise to guide a more thorough data analysis. Another path that this project can take is to investigate the maternal and fetal mortality disparity between private and public hospitals. These strategies may yield further associations and unveil implications that could provide insight into how the quality of healthcare and resources can be strategically enhanced to improve maternal and fetal outcomes across San Antonio and on a national level.

## Conclusions

The data suggest that there are disparities in healthcare between mothers residing in low and high SES areas in San Antonio, Texas. Women from the low SES ZIP Code had a greater incidence of non-routine discharges, multiple maternal-fetal risk factors, and fetal mortality, while women from the high SES ZIP Code experienced a greater incidence of fetal growth retardation. Further research is required to better understand the complex variables influencing maternal and fetal health, specifically in San Antonio. This research is meant to empower healthcare leaders to address disparities and enhance health outcomes across socioeconomic groups.

## Appendices

Appendix A

**Table 7:** Maternal Mortality in Texas, 2016 (N=245,364)

Characteristics	Patients, n (%)
Patient Status	
Routine Discharge	237,332 (96.7%)
Non-Routine Discharge	7,465 (3.0%)
Left Against Medical Advice	544 (0.2%)
Admitted	5 (>0.1%)
Expired	15 (>0.1%)
Still patient	3 (>0.1%)
Newborn affected by maternal death	0 (0.0%)
Death of unknown cause	3 (>0.1%)
Total maternal deaths	15 (0.01%)

Appendix B

**Table 8:** Maternal Discharge for all San Antonio Hospitals in 2016 (N=27,179)

Characteristics	Patients, n (%)
Patient Status	
Routine Discharge	27,022 (99.4%)
Non-Routine Discharge	118 (0.4%)
Left Against Medical Advice	38 (0.1%)
Admitted	1 (>0.1%)
Expired	0 (0.0%)
Still patient	0 (0.0%)
Newborn affected by maternal death	0 (0.0%)
Death of unknown cause	0 (0.0%)
Total maternal deaths	0 (0.0%)
